# Prospective Randomized Study Comparing Combined Phaco-ExPress and Phacotrabeculectomy in Open Angle Glaucoma Treatment: 12-Month Follow-Up

**DOI:** 10.1155/2015/720109

**Published:** 2015-06-07

**Authors:** Joanna Konopińska, Marta Deniziak, Emil Saeed, Agnieszka Bartczak, Renata Zalewska, Zofia Mariak, Marek Rękas

**Affiliations:** ^1^Department of Ophthalmology, Medical University of Białystok, M. Skłodowskiej-Curie 24A Street, 15-276 Białystok, Poland; ^2^Department of Ophthalmology, Military Institute of Medicine, Szaserów 128 Street, 04-141 Warsaw, Poland

## Abstract

*Purpose of the Study*. To compare the efficacy and safety of phacotrabeculectomy (P-Trab) and phacoemulsification with the ExPress (P-ExPress) mini glaucoma shunt implantation.* Study Plan*. Prospective randomized study.* Material and Methods*. 85 eyes with cataract and unregulated open angle glaucoma. There were 46 eyes in the P-ExPress and 39 the P-Trab group. Intraocular pressure (IOP), the number of antiglaucoma medications, qualified and complete surgical success (defined as IOP ≤ 18.0 mmHg), visual acuity (CDVA), the number of endothelial cells, and postoperative complications and additional procedures were assessed.* Results*. After 12 months of observation, the average IOP in the P-Express group went from 26.4 ± 9.3 down to 17.1 ± 5 mmHg (*P* < 0.05) and from 27.9 ± 12.9 down to 15.9 ± 2.7 mmHg in the P-Trab group (*P* < 0.05). No significant differences in the amount of medications used after surgery and CDVA were discovered between the groups. In the P-ExPress group, greater loss of endothelial cells was noted (CD_loss%_), compared to the P-Trab group.* Conclusions*. Both P-ExPress and P-Trab have comparable efficacy and similar early postoperative complication profile. The presence of additional implant (as is the case of the ExPress mini glaucoma shunt implantation) may cause progressive loss of endothelial cells.

## 1. Introduction

Trabeculectomy is the gold standard procedure used in the treatment of glaucoma, although its complication profile is quite broad [1, 2]. The ExPress mini glaucoma shunt implantation is one of the alternatives to this method. This method equals trabeculectomy in hypotensive effectiveness and is noted for less tissue traumatization, increased repeatability, and predictability of the hypotensive effect [3]. The safety profile of both procedures is similar [4, 5]. Although there are reports in the literature that paradoxically implant surgery may cause more corneal complications than trabeculectomy, generally it is considered to be a safer procedure [6]. According to* American Academy of Ophthalmology*, corneal endothelial decompensation [7] is a major implant-related complication. These reports induced the authors hereof to seek an answer to the question whether the ExPress mini glaucoma shunt may have a negative effect on the corneal endothelium in a long-term follow-up. The purpose of the study is to compare the combined antiglaucoma procedures, namely, phacoemulsification, with the ExPress mini glaucoma shunt implantation (the P-ExPress group) and phacotrabeculectomy (the P-Trab group) in terms of not only the hypotensive effect, but also the possibility of occurrence of complications related to cornea endothelial damage which may threaten the vision.

## 2. Material and Methods

### 2.1. Patients

The project is in line with the World Medical Association Declaration of Helsinki as well as the Principles of Good Clinical Practice developed by the European Union. It was approved by the Bioethics Committee at the Medical University in Białystok.

Coexisting glaucoma and cataract (NC1, NC2, and NC3) classified according to the LOCS III were an indication for surgery. Patients with primary open angle glaucoma (POAG), with secondary pseudoexfoliative glaucoma (PEX), and with pigmentary glaucoma (PG), in which satisfactory intraocular pressure (IOP > 21 mmHg) was not achieved despite maximally tolerated topical and systemic medication, were qualified for the treatment. Additional inclusion criteria were as follows: well-documented progression of visual field defects, significant diurnal variations in the IOP, lack of compliance to antiglaucoma therapy, and allergy to topical drugs. Written consent to participate in the study for at least 12 months was obtained from all patients after they were informed about the nature of the procedure and other surgical alternatives. Exclusion criteria were as follows: lack of consent to participate in the study, previous surgical and laser procedure within the eye, closed or narrow angle glaucoma, diabetes, advanced macular degeneration, and active inflammatory disease. Randomized prospective study included 85 eyes, of which 46 were subject to phacoemulsification with simultaneous ExPress mini glaucoma shunt implantation (P-ExPress), while in 39 eyes phacotrabeculectomy was performed. The toss of a coin was a method used to decide the assignment of a given patient into one of these study groups. Preoperative examination detailed data about previous treatments and surgical procedures were collected from the patients at the time of qualifications. Before surgical treatment, all patients underwent basic examination, which included determination of intraocular pressure (IOP), uncorrected distance visual acuity (UDVA), corrected distance visual acuity (CDVA), and examination of anterior and posterior segments of the eye. With specular microscope SP-3000P (Topcon Medical Systems 48 Inc., Oakland, USA), the central corneal thickness (CCT) and the number of endothelial cells per square millimetre (CD) were measured. On the basis of data obtained in different periods of observation, the CD_loss%_ was calculated, which represents the percentage loss of CD compared to baseline. In addition, the measurement of axial length of the eye (AXL) and keratometric parameters required for the intraocular lens calculation (IOL) were taken; furthermore, gonioscopy was performed and the field of view test was carried out (Humphrey SITA Standard 30-2). The IOP was measured during preoperative examination in accordance with AGIS. The measurement was taken using the slit lamp-mounted Goldmann applanation tonometer. The reading in mmHg was rounded to the next integer number. Each measurement was repeated twice, and if the difference between two readings was ≥ 3 mmHg, a third measurement was taken. The mean of two or three measurements was used to determine the IOP; it was also taken into account in statistical analysis. The IOP measurements were taken at the same time each day, that is, between 8 and 10 a.m. The IOL was calculated on the basis of the SRK T formula; furthermore, the anterior and posterior segments of the eye were assessed.

### 2.2. Surgical Technique

All surgical procedures were performed under retrobulbar anaesthesia (2% xylocaine and 0.5% bupivacaine) by two experienced surgeons (Zofia Mariak and Renata Zalewska). In both procedures, a fornix-based conjunctiva was dissected and the sclera was exposed. A limbus-based, square-shaped (4 × 4 mm) scleral flap was dissected using the technique previously described by Traverso et al. [5]. Then clear corneal incision for phacoemulsification was performed 2.75 mm from the temple with the phacochop technique using the Infiniti Vision System (Alcon Surgical, Fort Worth, TX) and an IOL was implanted. After phacoemulsification the cumulative dissipated energy (CDE) was recorded, and after implanting the WTK and rinsing the anterior chamber the aspiration time (AT) was registered. In the P-ExPress group, the mini glaucoma shunt was implanted for one hour, and in the P-Trab group, the trabeculectomy was performed using the technique described above [8]. The scleral flap was closed with the 10/0 nylon sutures (4 knotted sutures), whereas the conjunctiva was closed with absorbable sutures. Both during trabeculectomy and the ExPress mini glaucoma shunt implantation, 5-fluorouracil was used on a standard basis and applied in concentration of 50 mg/mL to the scleral wound bed for 3.5 minutes to avoid contact with the conjunctiva incision area [9].

### 2.3. Postoperative Protocol

During the follow-up visits, the IOL was measured using the Goldmann applanation tonometer, while corrected distance visual acuity (CDVA) was measured using the Snellen charts. All IOP measurements were taken between 8 and 10 a.m. The anterior chamber and the bottom of the eye were assessed. The postoperative period was assessed, including the complications and the number of antiglaucoma medications. Additional procedures were carried out when insufficient filtration was found; it was manifested in elevated IOP (≥16 mmHg) due to lack of drain through the shunt or underdeveloped or completely flat filtering bleb [9]. Inadequate filtration was diagnosed during the first two weeks after the surgery, when the healing process did not yet restrict the subconjunctival outflow and progressive increase in the IOP greater than 16 mmHg [9] was noticed. The presence and proper functioning of the filtering bleb and the development of subconjunctival fibrosis (manifested in overfull and tortuous blood vessels above the scleral flap) were assessed in detail. When the fibrosis was diagnosed based on the above symptoms, the subconjunctival outflow was insufficient, the IOP was increasing, and the bleb got flattened, the needling was performed. In the case of fibrosis, 5-FU subconjunctival injections were given (injection dose: 0.2 mL, 5 mg), combined with needling where applicable. Injections were given for 5 consecutive days or until the fibrosis abated and the IOP stabilized, provided that no antimetabolites-related adverse effects were encountered [10]. When flat, dysfunctional filtering bleb was noticed, the needling was performed. Suturolisis was performed within the first two weeks following the surgery, when poor filtration through the bleb was noticed (resulting from too tight scleral flap suturing). IOP ≤ 6 mmHg was considered to be hypotension.

The success rate was analyzed in two categories: complete and qualified. A complete surgical success rate was defined as IOP ≤ 18 mmHg without antiglaucoma medications, whereas qualified success rate was defined as IOP ≤ 18 mmHg with a maximum of two antiglaucoma medications. Failure was considered to have occurred when the IOP > 18 with or without antiglaucoma medications or when the eye required further surgical intervention. No antiglaucoma medications were allowed as of the day of the operation. When the operation did not produce the expected results, the medications were readministered as recommended by EGS. The control tests were carried out before the treatment and on the 1st and 7th postoperative days as well as on the 1st, 3rd, 6th, 9th, and 12th postoperative months. Visual field test and specular microscopy topography were carried out before the treatment and on the 6th and 12th postoperative months. Postoperatively, all patients were given antibiotic and steroid topically for 4 weeks. The Shapiro-Wilk test was used to assess the compliance of the parameters with normal distribution. For the one-way analysis of variance (ANOVA) purposes, the post hoc Bonferroni test was used in multiple comparisons or the Kruskal-Wallis test with Dunn's analysis of contrasts in the case of noncompliance of the parameters with normal distribution. The analysis between the groups was performed using Student's *t*-test or Mann-Whitney *U* test. The frequency table and Chi-squared test can be used for comparing quality characteristics. The significance level *P* < 0.05 was adopted for the purposes of calculations. Calculations were made using the SPSS and NCSS statistical packages.

## 3. Results

Forty-six patients underwent the P-ExPress implantation, while thirty-nine patients underwent phacotrabeculectomy. The average length of the follow-up period was 12.9 ± 0.4 months in the P-ExPress group and 12.7 ± 0.5 months in the P-Trab group. If reoperative surgery was considered necessary (due to complications or insufficient level of intraocular pressure), the patient was excluded from further participation in the study. Demographic data are summarized in Table 1.

### 3.1. Intraocular Pressure

**Figure 1:**
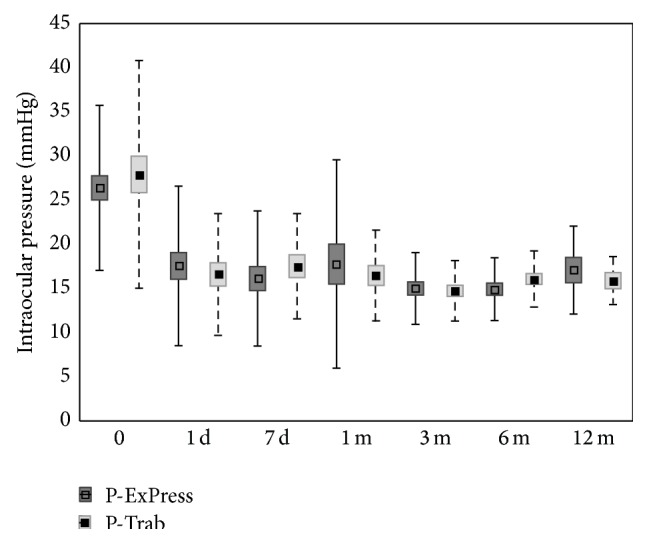
Mean values and standard deviations of intraocular pressure at specific times after surgery.

The average IOP before surgery in the P-ExPress group was 26.4 ± 9.3 mmHg; after 12 months of the follow-up, it decreased by 35.2% to 17.1 ± 5 mmHg (*P* < 0.05). In the P-Trab group, the average IOP was reduced from 27.9 ± 12.9 mmHg to 15.9 ± 2.7 mmHg (*P* < 0.05), which accounted for 43.0% reduction compared to baseline. Throughout the duration of the study, there were no statistically significant differences between the groups (*P* < 0.05) (Figure 1, Table 2).

### 3.2. Antiglaucoma Medications

Before surgery, the average number of antiglaucoma medications in the P-ExPress group was 2.9 ± 0.9, whereas after 12 months of the follow-up it went down to 1.25 ± 1.65 (*P* < 0.05). In the P-Trab group, the average number of medications went down from 3.4 ± 0.8 before operation to 1.2 ± 1.3 a year after surgery (*P* < 0.05). There were no statistical significant differences in the amount of medications between the groups before and after treatment (*P* > 0.05) (Table 3). Before surgery, 21.7% of the patients from the P-ExPress group and 21.6% of the patients from the P-Trab group were taking two antiglaucoma medications, while at the end of the follow-up period 50% and 75% of the patients from the P-ExPress and the P-Trab groups, respectively, did not take medications at all.

### 3.3. Surgical Success

**Figure 2:**
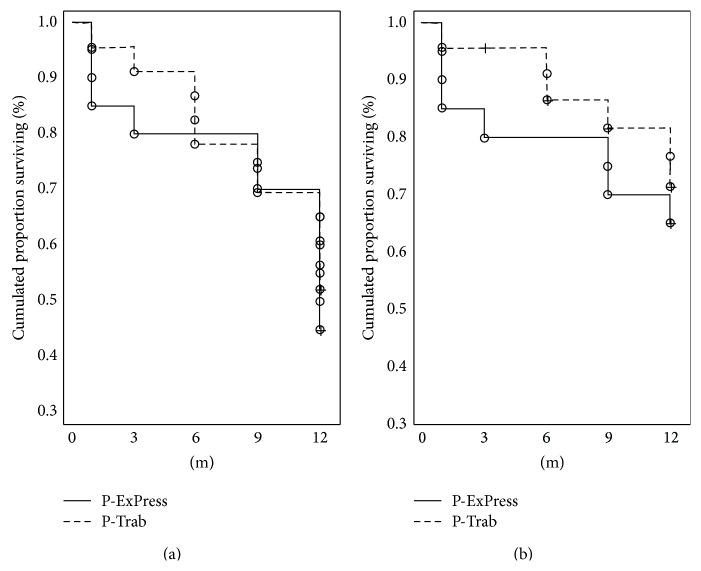
Cumulative surviving proportion (Kaplan-Meier) for success criterion of intraocular pressure less than or equal to 18 mmHg. (a) Without medications—complete success rate. (b) With max 2 or without medications—qualified success rate.

In the P-ExPress and the P-Trab groups, the complete success was achieved in 46% and 52% of the patients, respectively (*P* = 0.679), while qualified success was achieved in 65% of the cases in the P-ExPress group and in 72% of the cases in the P-Trab group (*P* = 0.556) (Figure 2).

### 3.4. Corrected Distance Visual Acuity

Before surgery, the CDVA in the P-ExPress group was 0.54 ± 0.56, whereas at the end of the follow-up period it improved and reached the value of 0.4 ± 0.54 (*P* < 0.05). In the P-Trab group, the CDVA was 0.34 ± 0.43 and 0.14 ± 0.18 (*P* < 0.05) before and after surgery, respectively. There were no significant differences between the groups after surgery throughout the entire follow-up period (*P* > 0.05) (Table 4).

At the end of the follow-up period, the loss of one Snellen line of vision was observed in 4 patients (8.7%) from the P-ExPress group. In 15.2% of the patients, the CDVA remained unchanged before and after surgery. 76.1% of the patients gained from 1 to 9 Snellen lines of the CDVA. The deterioration of vision in this group was caused by secondary cataract and macular oedema induced by chronic hypotension (one patient in each case). In the P-Trab group, the CDVA decreased by 1 Snellen line in 7 patients (17.9%); in 7.6% of the patients their visual acuity remained unchanged, while 74.3% of the patients gained from 1 to 9 Snellen lines of the CDVA. The leading causes of visual impairment included secondary cataract, epiretinal membrane, and dry AMD (age-related macular degeneration).

### 3.5. Visual Field

The mean defect (MD) before surgery was −17.0 ± 9.6 in the P-ExPress group and −7.9 ± 15.8 (*P* = 0.08) in the P-Trab group. However, after 12 months, it was −17.2 ± 10.4 and −14.8 ± 8.9 (*P* = 0.07), respectively. After 12 months, stabilization was observed in 83.3% of the patients from the P-ExPress group and in 69.2% (*P* = 0.07) of the patients from the P-Trab group. Progression was observed in 16.7% of the patients from the P-ExPress group and in 30.8% (*P* = 0.061) of the patients from the P-Trab group.

### 3.6. Endothelium

The average CCT before surgery amounted to 542.1 ± 31.9 *μ*m in the P-ExPress group and 540.0 ± 44.0 *μ*m in the P-Trab group (*P* = 0.817). Throughout the entire follow-up period, the average CCT did not change significantly and at the end of the follow-up period amounted to 525.1 ± 24.6 and 528.8 ± 27.6 in the P-ExPress and the P-Trab group, respectively (*P* = 0.602). Significant loss of endothelial cells in both groups (CD_loss%_) was observed in the follow-up period. After 12 months of the follow-up, the number of endothelial cells decreased by 37.4 ± 19.2% (*P* = 0.006) in the P-ExPress group and by 23.2 ± 14.1% (*P* = 0.008) in the P-Trab group. The difference between the groups was noted after 9 months following surgery and continued until the end of the follow-up period (Table 5).

### 3.7. Complications and Additional Procedures

Subconjunctival 5-fluorouracil injections were given to 9 patients from the P-ExPress group (19.6%) and 9 patients from the P-Trab group (23.1%) (*P* = 0.216). The average dose of 5-FU was 7.0 ± 3.5 mg in the P-ExPress group (1.4 injections on average) and 8.5 ± 2.3 mg in the P-Trab group (1.7 injections per patient on an average) (*P* = 0.105). In 11 patients from the P-ExPress group (23.9%) and in 12 patients from the P-Trab group (30.8%) (*P* = 0.305) the needling was performed. Laser suturolisis was performed in 1 patient from the P-ExPress group (2.1%) and in 2 patients from the P-Trab group (5.1%) (*P* = 0.462). One patient from the P-ExPress group was stitched up with additional seaming suture (*P* = 0.354). Two patients from the P-ExPress group underwent reoperation, one due to the mini shunt extrusion through the scleral flap and the other due to filtering bleb obstruction. In both cases, a classical trabeculectomy was performed. The patients were disqualified from the programme (their previous results were not excluded from the database). One patient from the P-Trab group underwent reoperation due to failure to stabilize the IOP. Malignant glaucoma symptoms, which occurred after 6 and 8 months after surgery, were observed in 1 patient. After applying cycloplegic drugs, the symptoms abated without additional surgical procedures (Table 6).

## 4. Discussion

The most commonly reported complications after trabeculectomy include bleeding into the anterior chamber, making the anterior chamber shallow and choroidal detachment [1]. The CDVA deterioration occurs in 30–50% of the cases [11] and surgery may achieve a success rate of 85%–90% [11, 12]. The ExPress mini glaucoma shunt implantation was proposed as a less-invasive procedure, with lower rate of complications resulting from postoperative hypotension. de Feo et al. reviewed postoperative test results in patients after the ExPress mini glaucoma shunt implementation and noted that the IOP decrease is comparable to the gold standard, that is, trabeculectomy, and emphasized lower risk of intra- and postoperative complications after the ExPress shunt implantation [13]. The ExPress mini glaucoma shunt is also used in eyes with advanced glaucoma, where it is necessary to maintain the target intraocular pressure at the lowest possible level [3]. Surgical success rate goes up to 87% [14]. Comparative study of these two techniques, conducted by Gallego-Pinazo et al., showed statistically lower IOP in the phacotrabeculectomy group in the early period of observation, with smaller percentage of occurrence of hypotension after the ExPress shunt implantation [15]. However, 6 and 15 months after the ExPress mini glaucoma shunt implantation, Maris Jr. et al. observed that the IOP was 13.7 ± 11.3 and 11.5 ± 11.1 mmHg, respectively, whereas in the trabeculectomy study group, it was 11.7 and 12.8 ± 8.7 ± 9.9 mmHg [16], respectively. In our study, 6 months after surgery, the IOP was 14.9 ± 3.6 in the P-ExPress group and 16.1 ± 3.2 mmHg in the P-Trab group (*P* = 0.281), and after 12 months it was 17.1 ± 5.0 and 15.9 ± 2.7 mmHg (*P* = 0.475), respectively. There were no significant differences in the IOP between the groups at any point of the follow-up period. The number of medications in the P-ExPress group decreased significantly from 2.91 ± 0.9 before surgery down to 1.25 ± 1.65 at the end of the follow-up period. As for the P-Trab group, the number of medications came to 3.26 ± 0.8 before and 1.2 ± 1.3 12 months after surgery. These results are consistent with the observations made by Coupin and Maris Jr. [14, 16]. In Dahan's study, in the group after the ExPress mini glaucoma shunt implantation, fewer patients needed antiglaucoma medications compared to the trabeculectomy group (0.3 versus 0.9) [8]. In both groups subject to the study, an improvement in visual acuity was observed in most patients; at the end of the follow-up period, in the P-ExPress group, it was 0.3 ± 0.49 versus 0.14 ± 0.18 logMAR in the P-Trab group. The complication profile in both groups was comparable and came to 45% and 56%, respectively. The most common complication in both groups was filtering bleb fibrosis (19.6% in the P-ExPress group and 23.1% in the P-Trab group). Other complications occurred at a similar rate to those reported in the literature (blood in the chamber: 2.1 versus 2.6%, leakage from the wound: 6.5 versus 2.6% versus 2.1, fixed hypotension: 2.6%, and choroidal detachment: 2.1 versus 7.7%). There were no statistically significant differences between the groups. No cases of the ExPress mini glaucoma shunt tip erosion through the scleral flap were noted. It is important to pay attention to the statistically significant difference in the CD and the CD_loss%_ observed in our study at the end of the follow-up period to the disadvantage of the P-ExPress group. As for the ExPress mini glaucoma shunts, their influence on the cornea condition depends on various factors such as the distance from the rear surface of the cornea, the material of which the implant is made, perioperative trauma, and the patient condition before surgery [17, 18]. In studies involving the use of animal models, it was noted that the material, of which the drainage device is made, affects the degree of cell loss [19]. Thus, in comparison with silicone and polymethylmethacrylate (PMMA), phosphorycholine polymer-coated PMMA (PC-PMMA) causes the least endothelial cells damage. There have been no reports in the literature showing the effect of stainless steel, of which the ExPress mini glaucoma shunt is made, on endothelial cells, whereas, in our analysis, the CD_loss%_ is significantly higher than in the case of implantation of an Ahmed glaucoma valve or Baerveldt glaucoma tube implant [20]. The exact mechanism damaging the endothelium by implants is not fully known. According to some theories, such damage is caused by increased fluid flow around the tip of the tube, inflammatory reaction in the anterior chamber, transitory contact between the tube and the cornea or between the tube and the uvea, or immune response evoked by the presence of a foreign body in the eye [21]. Other theories suggest that persistently elevated IOP directly or indirectly induces hypoxia, thereby damaging the endothelium [22]. Fiore et al. assume that the mechanism of endothelial damage may be associated with toxic effects of medications, preservatives contained in ophthalmic drops, and duration of treatment, making the anterior chamber shallow during and after operation or change of composition of fluid related to direct connection to the sub-Tenon's space [23]. Some researchers believe that patients taking three or four antiglaucoma medications at the same time have lower CD compared to those taking only one or two preparations [24]. In this study, we observed no statistically significant differences either in the IOP between the two groups or in terms of (i) average amount of medications taken before and after treatment and (ii) the number of patients not taking medications at all and undergoing monotherapy, polytherapy, and complex polytherapy. Another unfavourable factor is the use of antimetabolites during bleb-dependent surgeries [25, 26], although it is emphasized that they should not cause serious damage to the cornea unless they fall into the anterior chamber. Glaucoma surgical complications, often resulting from the cornea coming into contact with the iris, contribute to the reduction of endothelial cell density to a greater degree than complication-free operations; therefore, an accurate register of intra- and postoperative complications in the eyes subject to the study was kept. Statistical analysis showed no difference either in the amount of 5-FU given to both groups or in the number of complications between the groups. In the study called* Tube versus Trabeculectomy* (TVT), the efficacy of trabeculectomy and Baerveldt implant was compared [20]. Paradoxically, implantation turns out to cause more corneal complications than trabeculectomy, although generally it is considered to be a safer procedure. In patients who underwent Ahmed glaucoma valve implantation, time-related endothelial cells loss was observed [27–29]. The average CD after 12 months was 1910 ± 794, with the CD_loss%_ amounting to 16.6%. In our study, the CD at the end of the follow-up period was 1,337.2 ± 496.4, CD_loss%_  37.4 ± 19.2% (in the P-Express group) and 1427.6 ± 508.9, CD_loss%_  23.2 ± 14.1% (in the P-Trab group) (*P* = 0.691, *P* = 0.049). Such a difference between the findings may result from the fact that, in our case, the impact of the operation combined with simultaneous phacoemulsification is taken into account. Damage to endothelial cells after cataract surgery itself is documented [30–35]. Inasmuch as the impact of glaucoma shunts on the cornea has been fairly well studied, there are no long-lasting and repeatable findings on other operating techniques. Studies on the impact of trabeculectomy on endothelial cell density show great diversity: from 1.6% to 54.8% in studies conducted by Smith during the three-month follow-up period [36]. Arnavielle et al. [37] compared the endothelial cell loss after trabeculectomy and deep sclerectomy three months and one year after surgery. The researchers compared both single procedure and single procedure combined with cataract surgery. Statistically significant greater decrease in endothelial cells was found after trabeculectomy (CD_loss%_ 9.6%) and phacotrabeculectomy (CD_loss%_ 12.3%) compared to, respectively, sclerectomy (CD_loss%_ 4.5%) and phacosclerectomy (CD_loss%_ 7.8%). Shin et al. monitored the loss of endothelial cells in patients after trabeculectomy combined with mitomycin C for three months. They showed 7.7% decrease in endothelial cell density at the end of the follow-up period. It was, however, offset to 2.5% in the case of intraventricular administration of viscoelastic substance during surgery [38]. There are few studies of the endothelium condition after combined cataract and glaucoma surgeries (phacotrabeculectomies). Buys says that after three and twelve months, he noted much greater loss of endothelial cells in a group of patients who underwent cataract surgery and antiglaucoma surgery separately compared to those who underwent combined treatments [39]. After 24 months, these differences were not statistically significant any more. Our study has certain limitations such as short follow-up period and small control groups. Moreover, the results we obtained as regards the number of endothelial cells slightly exceed the threshold for statistical significance. Therefore, the above topic requires further studies with longer follow-up period and larger number of subjects.

## 5. Conclusions

The ExPress mini glaucoma shunt implantation is increasingly popular in surgical treatment of glaucoma as an alternative to traditional trabeculectomy because it is more predictable and allows the avoidance of many side effects. Hypotensive effect of both of these procedures is similar; however, it should be taken into account that the ExPress mini glaucoma shunt implantation has its own adverse effects profile, including corneal complications with significant, continued loss of endothelial cells in a greater extent than phacotrabeculectomy.

## Figures and Tables

**Table 1:** Patients' demographic data.

Group	P-ExPress	P-Trab	*P* ^*∗*^
Follow-up (months)	12.9 ± 0.4	12.7 ± 0.5	0.351
*n*	46	39	—
Age (years)	71.8 ± 9.46	69.6 ± 10.83	0.487
Sex (female/male)	29/17	29/10	0.579
Eye (right/left)	25/21	17/22	0.856
Glaucoma type			
POAG	29	26	0.468
PEX	15	13	
Pigmentary	2	0	
LOCS III scale (NC_1_/NC_2_/NC_3_)	14/28/4	10/24/5	0.899
CDE	5.21 ± 0.27	5.78 ± 0.37	0.532
AT	1.31 ± 0.22	1.24 ± 0.47	0.401

P-ExPress: phaco-ExPress group, P-Trab: phacotrabeculectomy group; ^*∗*^Student's *t*-test or *χ* ^2^ test; POAG: primary open angle glaucoma; PEX: pseudoexfoliation glaucoma; LOCS III: lens opacities classification system III; CDE: cumulative dissipated energy; AT: aspiration time (min).

**Table 2:** Intraocular pressure (IOP) mean values, median values, standard deviations, and range in the phaco-ExPress (P-ExPress) and phacotrabeculectomy (P-Trab) groups at specific times after surgery.

Time	P-ExPress			P-Trab			*P* ^*∗*^
	Mean (SD)	Median	Range	Mean (SD)	Median	Range	
Pre-op	26.4 ± 9.3	25.00	12–50	27.9 ± 12.9	25.00	12–62	0.877
1st day	17.6 ± 9.0	16.00	4–29	16.6 ± 6.9	16.00	3–31	0.867
7th day	16.1 ± 7.6	14.00	7–23	17.5 ± 5.9	18.00	3–19	0.236
1st month	17.8 ± 11.8	16.00	8–27	16.5 ± 5.1	16.00	10–26	0.653
3rd month	15.0 ± 4.1	16.00	5–23	14.7 ± 3.4	15.00	7–24	0.645
6th month	14.9 ± 3.6	15.00	7–22	16.1 ± 3.2	16.00	8–23	0.281
9th month	15.8 ± 6.6	17.00	9–26	15.1 ± 3.2	15.00	9–25	0.318
12th month	17.1 ± 5.0	17.50	7–26	15.9 ± 2.7	16.00	8–26	0.475

P-ExPress: phaco-ExPress group; P-Trab: phacotrabeculectomy group; SD: standard deviation; Pre-op: preoperatively, ^*∗*^Mann-Whitney *U* test.

**Table 3:** Amount of hypotensive drugs: mean values, median values, standard deviations, and range in the phaco-ExPress (P-ExPress) and phacotrabeculectomy (P-Trab) groups at specific times after surgery.

Time	P-ExPress			P-Trab			*P* ^*∗*^
	Mean (SD)	Median	Range	Mean (SD)	Median	Range	
Pre-op	2.91 ± 0.9	3.0	2–4	3.26 ± 0.8	3.0	3-4	0.079
1st day	0.1 ± 0.5	0	—	0.2 ± 0.6	0	—	0.540
7th day	0.06 ± 0.3	0	—	0.06 ± 0.3	0	—	0.968
1st month	0.15 ± 0.5	0	—	0.07 ± 0.4	0	—	0.535
3rd month	0.27 ± 0.6	0	—	0.37 ± 0.7	0	—	0.585
6th month	0.46 ± 1	0	—	1.39 ± 1.23	2	0–2	0.003
9th month	1 ± 1.6	0	0–2.25	1.35 ± 1	2	0–2	0.177
12th month	1.25 ± 1.65	0	0–2.75	1.2 ± 1.3	1	0–2	0.919

Pre-op: preoperatively. ^*∗*^Mann-Whitney *U* test.

**Table 4:** Visual acuity (logMAR) mean values, median values, standard deviations, and range in the phaco-ExPress (P-ExPress) and phacotrabeculectomy (P-Trab) groups at specific times after surgery.

Time	P-ExPress			P-Trab			*P* ^*∗*^
	Mean (SD)	Median	Range	Mean (SD)	Median	Range	
Pre-op	0.54 ± 0.56	0.30	0–2.4	0.34 ± 0.43	0.22	0–2	0.046
1st day	0.81 ± 0.58	0.70	0–2	0.53 ± 0.49	0.30	0.05–2	0.037
7th day	0.43 ± 0.52	0.22	0–2	0.39 ± 0.45	0.22	0–2	0.780
1st month	0.38 ± 0.45	0.22	0–1.7	0.34 ± 0.49	0.15	0–2	0.621
3rd month	0.24 ± 0.44	0.12	0–1.7	0.18 ± 0.29	0.07	0–1.4	0.570
6th month	0.24 ± 0.45	0.05	0–1.7	0.2 ± 0.3	0.13	0–1.4	0.530
9th month	0.4 ± 0.54	0.13	0.0–1.7	0.18 ± 0.31	0.10	0–1.4	0.863
12th month	0.3 ± 0.49	0.22	0–1.7	0.14 ± 0.18	0.02	0–0.4	0.259

P-ExPress: phaco-ExPress group; P-Trab: phacotrabeculectomy group; SD: standard deviation; Pre-op: preoperatively, ^*∗*^Mann-Whitney *U* test.

**Table 5:** Corneal epithelial cells density CD, CD_loss%_: mean values, median values, standard deviations, and range in the P-ExPress and P-Trab groups at specific times after surgery.

Parameter	P-ExPress group			P-Trab group			*P* ^*∗*^
	Mean (SD)	Median	Range	Mean (SD)	Median	Range	
CD_0_	2089.3 ± 421.6	2135	1784–2374	1985.7 ± 545.5	2059	1537–2450	0.370
CD_6 m_	1574.8 ± 479.2	1591	1178–1866	1594.3 ± 499.7	1653	1155–2009	0.883
CD_6 m-loss%_	21.4 ± 16.7	18.4	8.84–31.38	19.7 ± 14.1	17	8.18 ± 26.9	0.677
CD_9 m_	1355.8 ± 434.7	1389	894–1714	1551.5 ± 481.8	1543	1073–1928	0.047
CD_9 m-loss%_	36.2 ± 20.7	34.5	19.47–48.5	21.7 ± 12.3	20.1	12.75–28.8	0.008
CD_12 m_	1337.2 ± 496.4	1430	1053–1721	1427.6 ± 508.9	1310	961–1938	0.691
CD_12 m-loss%_	37.4 ± 19.2	36.1	22.67–48.2	23.2 ± 14.1	22.6	16.01–27.6	0.049

P-ExPress: phaco-ExPress group; P-Trab: phacotrabeculectomy group; SD: standard deviation; CD: epithelial cells density; CD_loss%_: epithelial cells density loss %, ^*∗*^Mann-Whitney *U* test.

**Table 6:** Postoperative complications.

Complications	P-ExPress *n* (%)	P-Trab *n* (%)	*P* ^*∗*^
Intraoperative			
Bleeding	—	1 (2.6)	0.645
Postoperative			
Hyphema			
Blood level in AC	1 (2.1)	1 (2.6)	0.875
Erythrocytes in AC	—	—	—
Wound leakage	3 (6.5)	1 (2.6)	0.391
Fibrosis	9 (19.6)	9 (23.1)	0.784
Anterior chamber cells	3 (6.5)	3 (7.7)	0.834
Hypotony			
Until 7 days	—	2 (5.1)	0.115
Until 30 days	—	1 (2.6)	0.411
Until 180 days	1 (2.1)	1 (2.6)	0.896
Choroid detachment	1 (2.1)	3 (7.7)	0.231
Macular edema	1 (2.1)	—	0.354
Malignant glaucoma	2 (4.3)	—	0.187

P-ExPress: phaco-ExPress group; P-Trab: phacotrabeculectomy group; ^*∗*^ *χ* ^2^ test.
